# Supplementary material for: Long-term trends in death and dependence after ischaemic strokes: A retrospective cohort study using the South London Stroke Register (SLSR)
Source: PLoS Med. 2020 Mar 12;17(3):e1003048. doi: 10.1371/journal.pmed.1003048 (PMC7067375; doi:10.1371/journal.pmed.1003048)
Supplement: S1 Appendix — (PDF) [file pmed.1003048.s002.pdf]

Supplementary Appendix

Long–term trends in death and dependence after ischaemic strokes: a retrospective cohort study using the South London stroke register (SLSR)

Hatem A Wafa, Charles D.A. Wolfe, Ajay Bhalla, Yanzhong Wang

TABLE OF CONTENT

Table A: Survival models for ISs, overall and by ethnic groups 6
Table B: Survival models for ISs by sex groups 8
Table C: Survival models for ISs by age groups 10
Table D: Trends in medication use among patients with IS 11
Table E: Medication use among patients with ISs by aetiological subtypes 11
Table F: Differences between IS patients with complete BI at 3 months and those without .12
Table G: Trends in functional dependence (mRS ≥3) 13

Fig A: Survival after IS stroke by TOAST aetiological subtypes, and age, sex, and ethnic groups...3
Fig B: Time–trends in survival after IS stroke by TOAST aetiological subtypes.....................3
Fig C: Time–trends in survival after IS stroke by sex groups...4
Fig D: Time–trends in survival after IS stroke by ethnic groups 4
Fig E: Time–trends in survival after IS stroke by age groups...4

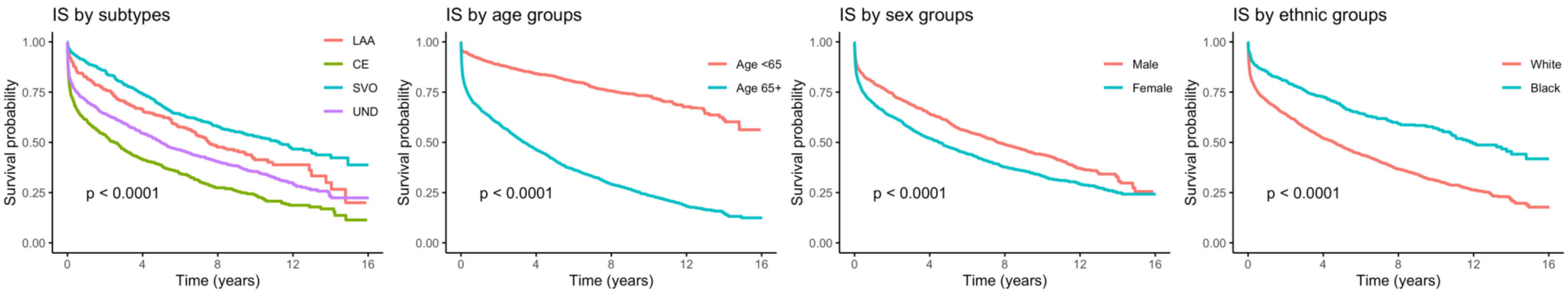

Fig A: Survival after IS stroke by TOAST aetiological subtypes, and age, sex, and ethnic groups.

P values were obtained from the log–rank tests (unadjusted).

Abbreviations: CE, cardio–embolism; IS, ischaemic stroke; LAA, large–artery atherosclerosis; SVO, small–vessel occlusion; TOAST, Trial of ORG 10172 in Acute Stroke Treatment; UND, undetermined aetiologies.

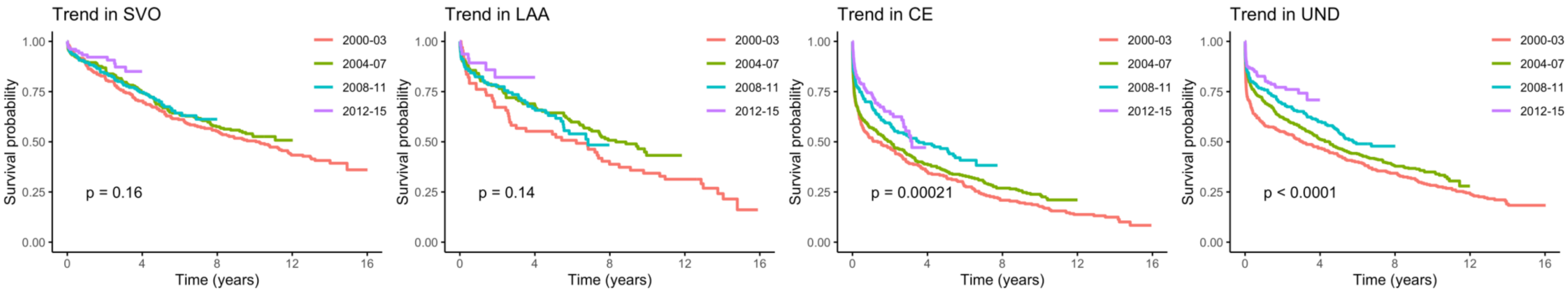

Fig B: Time–trends in survival after IS stroke by TOAST aetiological subtypes.

P values were obtained from the log–rank tests (unadjusted).

Abbreviations: CE, cardio–embolism; LAA, large–artery atherosclerosis; SVO, small–vessel occlusion; TOAST, Trial of ORG 10172 in Acute Stroke Treatment; UND, undetermined aetiologies.

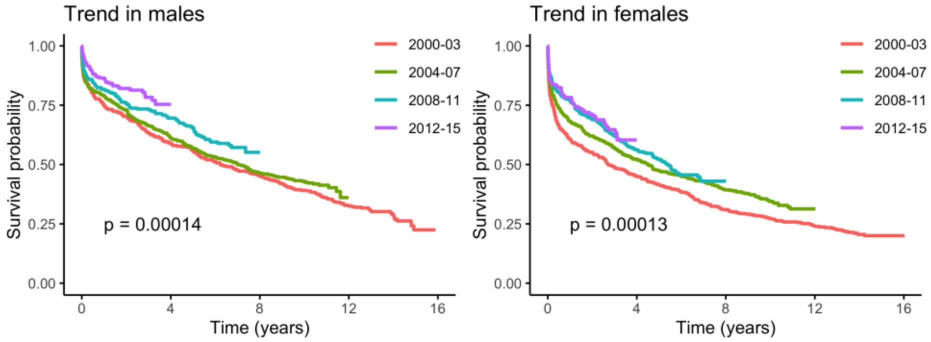

Fig C: Time–trends in survival after IS stroke by sex groups.

P values were obtained from the log–rank tests (unadjusted).

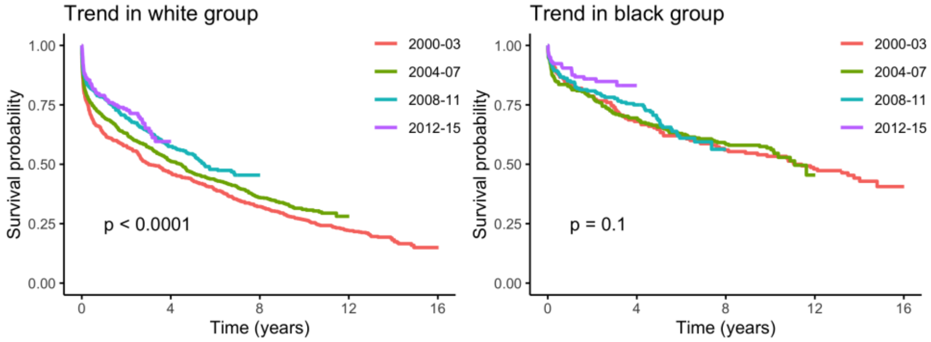

Fig D: Time–trends in survival after IS stroke by ethnic groups.

P values were obtained from the log–rank tests (unadjusted).

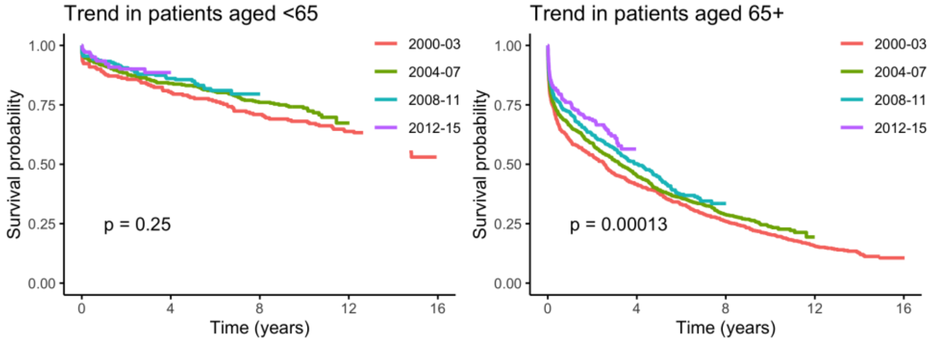

Fig E: Time–trends in survival after IS stroke by age groups.

P values were obtained from the log–rank tests (unadjusted).

	Overall		White		Black	
	HR (95% CI)	P Value	HR (95% CI)	P Value	HR (95% CI)	P Value
**All ischaemic strokes (ISs)**						
Time (year)	0.976 (0.959–0.993)	0.006*	0.978 (0.96–0.995)	0.013*	0.962 (0.929–0.996)	0.028*
Age	1.063 (1.057–1.068)	<0.0001*	1.066 (1.059–1.073)	<0.0001*	1.059 (1.048–1.071)	<0.0001*
Female (vs male)	0.864 (0.775–0.962)	0.008*	0.845 (0.744–0.96)	0.01*	0.832 (0.648–1.068)	0.15
Black (vs white)	0.836 (0.658–1.061)	0.14				
Other (vs white)	0.775 (0.55–1.094)	0.15				
Current drinker	0.843 (0.754–0.942)	0.003*	0.831 (0.732–0.944)	0.004*	0.804 (0.608–1.064)	0.13
Smoker	1.533 (1.35–1.74)	<0.0001*	1.482 (1.279–1.716)	<0.0001*	1.813 (1.301–2.527)	0.0005*
Hypertension	0.967 (0.862–1.085)	0.57	1.023 (0.9–1.163)	0.73	0.698 (0.51–0.957)	0.025*
Diabetes mellitus	1.273 (1.124–1.442)	0.0001*	1.255 (1.078–1.461)	0.003*	1.355 (1.044–1.758)	0.023*
Hypercholesterolaemia	0.939 (0.829–1.063)	0.32	0.89 (0.77–1.027)	0.11	1.106 (0.819–1.493)	0.51
Atrial fibrillation	1.372 (1.205–1.562)	<0.0001*	1.278 (1.109–1.474)	0.0007*	1.803 (1.225–2.654)	0.003*
Myocardial infarction	1.231 (1.055–1.437)	0.008*	1.267 (1.064–1.509)	0.008*	1.446 (0.937–2.231)	0.1
TIA	1.098 (0.945–1.277)	0.22	1.029 (0.868–1.22)	0.74	1.418 (0.926–2.17)	0.11
Stroke unit admission (vs none)	1.069 (0.869–1.314)	0.53	1.079 (0.849–1.37)	0.53	1.351 (0.801–2.28)	0.26
Other medical wards (vs none)	1.436 (1.158–1.78)	0.001*	1.392 (1.087–1.784)	0.009*	1.473 (0.826–2.627)	0.19
Anticoagulant	0.952 (0.802–1.13)	0.58	0.983 (0.814–1.187)	0.86	0.862 (0.559–1.329)	0.5
Antiplatelet	0.659 (0.542–0.801)	0.0001*	0.696 (0.558–0.869)	0.002*	0.539 (0.338–0.86)	0.011*
Thrombolysis	0.817 (0.655–1.02)	0.08	0.785 (0.606–1.016)	0.07	0.824 (0.492–1.381)	0.46
GCS	0.923 (0.902–0.944)	<0.0001*	0.927 (0.903–0.953)	<0.0001*	0.923 (0.869–0.98)	0.009*
Failed swallowing	1.318 (1.147–1.515)	0.0001*	1.283 (1.097–1.501)	0.002*	1.319 (0.912–1.907)	0.14
Urinary incontinence	1.199 (1.042–1.38)	0.012*	1.106 (0.943–1.297)	0.21	1.495 (1.059–2.112)	0.023*
BI (7d)	0.943 (0.931–0.955)	<0.0001*	0.941 (0.928–0.955)	<0.0001*	0.943 (0.917–0.969)	<0.0001*
NIHSS	1.037 (1.025–1.049)	<0.0001*	1.038 (1.024–1.052)	<0.0001*	1.033 (1.004–1.063)	0.026*
**Large artery atherosclerosis (LAA)**						
Time (year)	0.979 (0.923–1.037)	0.47	0.968 (0.91–1.029)	0.29	0.917 (0.702–1.198)	0.53
Age	1.081 (1.062–1.1)	<0.0001*	1.075 (1.052–1.099)	<0.0001*	1.15 (1.053–1.256)	0.002*
Female (vs male)	0.887 (0.624–1.263)	0.51	0.968 (0.639–1.467)	0.88	0.734 (0.203–2.65)	0.64
Black (vs white)	0.772 (0.32–1.861)	0.56				
Other (vs white)	0.297 (0.089–0.991)	0.048*				
Current drinker	0.846 (0.579–1.235)	0.39	0.84 (0.558–1.266)	0.4	0.58 (0.098–3.419)	0.55
Smoker	1.643 (1.091–2.475)	0.018*	1.531 (0.943–2.487)	0.09	5.208 (0.727–37.316)	0.1
Hypertension	1.3 (0.889–1.9)	0.18	1.332 (0.869–2.042)	0.19	3.143 (0.334–29.529)	0.32
Diabetes mellitus	1.777 (1.154–2.735)	0.009*	1.568 (0.894–2.75)	0.12	12.807 (1.858–88.281)	0.011*
Hypercholesterolaemia	0.563 (0.362–0.875)	0.011*	0.54 (0.322–0.907)	0.02*	0.404 (0.104–1.578)	0.19
Atrial fibrillation	1.798 (0.757–4.267)	0.18	1.82 (0.717–4.617)	0.21	32.452 (1.113–946.419)	0.044*
Myocardial infarction	1.72 (0.988–2.997)	0.06	1.548 (0.8–2.997)	0.19	3.126 (0.504–19.381)	0.22
TIA	1.184 (0.753–1.862)	0.47	1.194 (0.717–1.99)	0.5	3.575 (0.584–21.88)	0.17
Stroke unit admission (vs none)	1.105 (0.503–2.424)	0.8	1.074 (0.465–2.482)	0.87	9782218.845 (0–Inf)	1
Other medical wards (vs none)	1.28 (0.542–3.027)	0.57	1.129 (0.449–2.842)	0.8	16915675.158 (0–Inf)	1
Anticoagulant	0.886 (0.498–1.576)	0.68	0.867 (0.449–1.674)	0.67	0.301 (0.018–5.104)	0.41
Antiplatelet	0.645 (0.363–1.145)	0.14	0.709 (0.373–1.347)	0.29	0.453 (0.015–13.426)	0.65
Thrombolysis	0.784 (0.436–1.412)	0.42	0.766 (0.368–1.593)	0.48	0.508 (0.051–5.052)	0.56
GCS	0.8 (0.712–0.9)	0.0002*	0.803 (0.697–0.926)	0.003*	0.698 (0.46–1.058)	0.09
Failed swallowing	0.977 (0.62–1.541)	0.92	0.887 (0.517–1.521)	0.66	2.468 (0.305–20.006)	0.4
Urinary incontinence	1.301 (0.825–2.053)	0.26	1.241 (0.731–2.108)	0.42	0.898 (0.111–7.258)	0.92
BI (7d)	0.925 (0.883–0.97)	0.001*	0.915 (0.866–0.966)	0.002*	0.963 (0.82–1.132)	0.65
NIHSS	1.055 (1.007–1.105)	0.024*	1.043 (0.987–1.102)	0.14	1.204 (1.001–1.448)	0.05
**Cardio–embolism (CE)**						
Time (year)	0.972 (0.945–0.998)	0.034*	0.97 (0.943–0.998)	0.036*	0.946 (0.876–1.022)	0.16
Age	1.058 (1.048–1.068)	<0.0001*	1.064 (1.051–1.077)	<0.0001*	1.052 (1.03–1.074)	<0.0001*
Female (vs male)	0.828 (0.679–1.009)	0.06	0.73 (0.579–0.92)	0.008*	0.928 (0.54–1.596)	0.79
Black (vs white)	1.218 (0.777–1.911)	0.39				
Other (vs white)	1.049 (0.572–1.927)	0.88				
Current drinker	0.789 (0.644–0.967)	0.023*	0.725 (0.575–0.915)	0.007*	0.686 (0.367–1.28)	0.24
Smoker	1.777 (1.368–2.309)	<0.0001*	1.723 (1.284–2.314)	0.0003*	2.565 (0.938–7.013)	0.07
Hypertension	0.98 (0.798–1.203)	0.85	1.094 (0.868–1.379)	0.45	0.694 (0.313–1.542)	0.37
Diabetes mellitus	1.14 (0.893–1.454)	0.29	1.154 (0.867–1.536)	0.33	1.128 (0.588–2.165)	0.72
Hypercholesterolaemia	0.942 (0.754–1.177)	0.6	0.852 (0.666–1.09)	0.2	1.881 (0.919–3.85)	0.08
Atrial fibrillation	1.217 (1.005–1.473)	0.044*	1.162 (0.94–1.436)	0.17	1.216 (0.654–2.26)	0.54
Myocardial infarction	1.09 (0.855–1.39)	0.49	1.061 (0.807–1.394)	0.67	1.908 (0.828–4.395)	0.13
TIA	1.095 (0.837–1.433)	0.51	1.064 (0.786–1.439)	0.69	1.199 (0.488–2.946)	0.69

Stroke unit admission (vs none)	0.782 (0.493–1.241)	0.3	0.755 (0.45–1.264)	0.28	3.232 (0.702–14.878)	0.13
Other medical wards (vs none)	1.226 (0.766–1.961)	0.4	1.077 (0.638–1.819)	0.78	3.98 (0.752–21.069)	0.1
Anticoagulant	0.908 (0.715–1.154)	0.43	1.034 (0.77–1.39)	0.82	0.445 (0.195–1.016)	0.06
Antiplatelet	0.694 (0.476–1.012)	0.06	0.783 (0.512–1.197)	0.26	0.377 (0.17–0.837)	0.017*
Thrombolysis	0.808 (0.573–1.14)	0.23	0.928 (0.627–1.374)	0.71	0.289 (0.084–0.988)	0.049*
GCS	0.943 (0.906–0.982)	0.004*	0.94 (0.898–0.983)	0.007*	1.012 (0.895–1.146)	0.84
Failed swallowing	1.146 (0.881–1.489)	0.31	1.152 (0.864–1.536)	0.34	1.058 (0.463–2.42)	0.89
Urinary incontinence	1.248 (0.952–1.636)	0.11	1.111 (0.807–1.53)	0.52	1.244 (0.582–2.659)	0.57
BI (7d)	0.948 (0.927–0.969)	<0.0001*	0.95 (0.927–0.974)	<0.0001*	0.925 (0.869–0.985)	0.015*
NIHSS	1.036 (1.016–1.056)	0.0004*	1.034 (1.011–1.057)	0.004*	1.056 (1–1.115)	0.05*
**Small vessel occlusion (SVO)**						
Time (year)	0.96 (0.917–1.006)	0.08	0.956 (0.91–1.004)	0.07	0.944 (0.87–1.025)	0.17
Age	1.065 (1.051–1.079)	<0.0001*	1.067 (1.051–1.084)	<0.0001*	1.056 (1.025–1.088)	0.0003*
Female (vs male)	0.882 (0.692–1.123)	0.31	0.914 (0.677–1.233)	0.55	0.934 (0.542–1.61)	0.81
Black (vs white)	0.697 (0.427–1.137)	0.15				
Other (vs white)	0.704 (0.328–1.514)	0.37				
Current drinker	0.809 (0.626–1.045)	0.1	0.797 (0.575–1.105)	0.17	0.961 (0.549–1.68)	0.89
Smoker	1.48 (1.116–1.962)	0.007*	1.365 (0.958–1.946)	0.09	1.622 (0.842–3.123)	0.15
Hypertension	0.88 (0.676–1.145)	0.34	0.925 (0.68–1.259)	0.62	0.425 (0.216–0.837)	0.013*
Diabetes mellitus	1.31 (0.99–1.734)	0.06	1.019 (0.69–1.506)	0.92	1.592 (0.886–2.859)	0.12
Hypercholesterolaemia	1.129 (0.846–1.506)	0.41	1.005 (0.705–1.434)	0.98	2.117 (1.068–4.194)	0.032*
Atrial fibrillation	2.084 (1.264–3.435)	0.004*	2.267 (1.185–4.337)	0.014*	2.941 (1.015–8.524)	0.047*
Myocardial infarction	1.587 (1.018–2.475)	0.042*	2.155 (1.217–3.817)	0.008*	1.19 (0.427–3.315)	0.74
TIA	1.073 (0.728–1.582)	0.72	0.902 (0.557–1.46)	0.67	2.759 (1.183–6.436)	0.019*
Stroke unit admission (vs none)	1.456 (1.004–2.112)	0.048*	1.296 (0.832–2.02)	0.25	1.598 (0.653–3.914)	0.3
Other medical wards (vs none)	1.356 (0.882–2.083)	0.17	1.073 (0.639–1.8)	0.79	1.312 (0.495–3.477)	0.58
Anticoagulant	1.062 (0.654–1.724)	0.81	1.114 (0.622–1.995)	0.72	0.551 (0.152–2.003)	0.37
Antiplatelet	0.577 (0.38–0.875)	0.011*	0.722 (0.433–1.202)	0.21	0.452 (0.132–1.547)	0.21
Thrombolysis	0.949 (0.366–2.461)	0.91	0.822 (0.246–2.743)	0.75	0.828 (0.065–10.533)	0.88
GCS	0.973 (0.907–1.044)	0.45	0.997 (0.908–1.095)	0.95	0.916 (0.745–1.126)	0.4
Failed swallowing	1.544 (1.086–2.194)	0.016*	1.683 (1.112–2.546)	0.014*	1.222 (0.47–3.179)	0.68
Urinary incontinence	1.142 (0.835–1.561)	0.4	1.172 (0.806–1.706)	0.41	1.815 (0.782–4.214)	0.17
BI (7d)	0.935 (0.91–0.961)	<0.0001*	0.938 (0.908–0.969)	0.0001*	0.923 (0.863–0.987)	0.02*
NIHSS	1.018 (0.978–1.06)	0.38	1.018 (0.97–1.069)	0.47	1.023 (0.928–1.127)	0.65
**Undetermined aetiologies (UND)**						
Time (year)	0.985 (0.955–1.016)	0.34	0.991 (0.96–1.024)	0.59	0.951 (0.897–1.008)	0.09
Age	1.061 (1.052–1.071)	<0.0001*	1.066 (1.054–1.078)	<0.0001*	1.063 (1.042–1.085)	<0.0001*
Female (vs male)	0.842 (0.698–1.016)	0.07	0.837 (0.672–1.042)	0.11	0.907 (0.569–1.448)	0.68
Black (vs white)	0.939 (0.615–1.435)	0.77				
Other (vs white)	0.612 (0.325–1.149)	0.13				
Current drinker	0.92 (0.754–1.122)	0.41	0.923 (0.739–1.153)	0.48	0.933 (0.547–1.591)	0.8
Smoker	1.548 (1.262–1.9)	<0.0001*	1.558 (1.224–1.981)	0.0003*	2.121 (1.228–3.663)	0.007*
Hypertension	0.941 (0.782–1.132)	0.52	0.925 (0.751–1.139)	0.46	1.111 (0.645–1.915)	0.7
Diabetes mellitus	1.176 (0.958–1.444)	0.12	1.345 (1.052–1.719)	0.018*	1.002 (0.624–1.608)	0.99
Hypercholesterolaemia	1.024 (0.829–1.266)	0.82	1.051 (0.816–1.354)	0.7	0.673 (0.39–1.161)	0.15
Atrial fibrillation	1.204 (0.949–1.528)	0.13	1.072 (0.826–1.392)	0.6	2.429 (1.048–5.626)	0.039*
Myocardial infarction	1.061 (0.801–1.406)	0.68	1.158 (0.838–1.599)	0.37	1.061 (0.402–2.798)	0.91
TIA	1.227 (0.949–1.586)	0.12	1.199 (0.905–1.588)	0.21	1.781 (0.697–4.552)	0.23
Stroke unit admission (vs none)	0.983 (0.682–1.419)	0.93	1.128 (0.727–1.751)	0.59	1.048 (0.411–2.673)	0.92
Other medical wards (vs none)	1.441 (0.996–2.086)	0.05	1.67 (1.074–2.598)	0.023*	1.203 (0.397–3.645)	0.74
Anticoagulant	0.893 (0.653–1.22)	0.48	0.858 (0.61–1.206)	0.38	1.37 (0.584–3.212)	0.47
Antiplatelet	0.634 (0.475–0.847)	0.003*	0.616 (0.438–0.867)	0.007*	0.52 (0.225–1.198)	0.13
Thrombolysis	0.798 (0.554–1.149)	0.23	0.655 (0.419–1.026)	0.07	1.069 (0.491–2.326)	0.87
GCS	0.895 (0.858–0.934)	<0.0001*	0.891 (0.843–0.942)	<0.0001*	0.867 (0.785–0.958)	0.006*
Failed swallowing	1.434 (1.148–1.79)	0.002*	1.475 (1.145–1.901)	0.003*	1.275 (0.659–2.467)	0.47
Urinary incontinence	1.253 (0.996–1.575)	0.05	1.093 (0.832–1.435)	0.52	2.293 (1.267–4.149)	0.006*
BI (7d)	0.942 (0.925–0.96)	<0.0001*	0.936 (0.915–0.957)	<0.0001*	0.942 (0.902–0.984)	0.007*
NIHSS	1.035 (1.016–1.055)	0.0005*	1.045 (1.02–1.07)	0.0005*	0.989 (0.939–1.041)	0.67

Table A: Survival models for ISs, overall and by ethnic groups.

***** Denotes significant trends (*p* <0.05).

Abbreviations: BI, Barthel index; CI, confidence interval; GCS, Glasgow coma scale; NIHSS, National Institutes of Health Stroke Scale; and TIA, transient ischaemic attack.

	Males		Females	
	HR (95% CI)	P Value	HR (95% CI)	P Value
**All ischaemic strokes (ISs)**				
Time (year)	0.975 (0.953–0.998)	0.034*	0.976 (0.956–0.995)	0.016*
Age	1.066 (1.058–1.074)	<0.0001*	1.061 (1.053–1.069)	<0.0001*
Black (vs white)	0.816 (0.672–0.991)	0.04*	0.835 (0.681–1.023)	0.08
Other (vs white)	0.771 (0.579–1.026)	0.07	0.964 (0.728–1.276)	0.8
Current drinker	0.855 (0.721–1.015)	0.07	0.822 (0.705–0.96)	0.013*
Smoker	1.536 (1.282–1.841)	<0.0001*	1.564 (1.279–1.913)	<0.0001*
Hypertension	0.961 (0.814–1.134)	0.64	0.982 (0.838–1.149)	0.82
Diabetes mellitus	1.417 (1.183–1.696)	0.0002*	1.154 (0.971–1.371)	0.1
Hypercholesterolaemia	0.978 (0.813–1.176)	0.81	0.922 (0.776–1.094)	0.35
Atrial fibrillation	1.362 (1.11–1.671)	0.003*	1.375 (1.169–1.618)	0.0001*
Myocardial infarction	1.047 (0.837–1.31)	0.69	1.468 (1.172–1.839)	0.0008*
TIA	1.005 (0.793–1.274)	0.97	1.151 (0.94–1.409)	0.17
Stroke unit admission (vs none)	1.204 (0.897–1.615)	0.22	0.956 (0.712–1.283)	0.76
Other medical wards (vs none)	1.775 (1.298–2.427)	0.0003*	1.204 (0.892–1.626)	0.23
Anticoagulant	0.909 (0.702–1.178)	0.47	1.014 (0.804–1.277)	0.91
Antiplatelet	0.628 (0.476–0.828)	0.002*	0.684 (0.537–0.87)	0.003*
Thrombolysis	0.864 (0.634–1.178)	0.36	0.794 (0.602–1.048)	0.11
GCS	0.942 (0.91–0.975)	0.0007*	0.903 (0.875–0.933)	<0.0001*
Failed swallowing	1.41 (1.14–1.745)	0.002*	1.267 (1.049–1.53)	0.014*
Urinary incontinence	1.24 (1.006–1.529)	0.044*	1.137 (0.933–1.384)	0.2
BI (7d)	0.955 (0.938–0.972)	<0.0001*	0.931 (0.915–0.946)	<0.0001*
NIHSS	1.041 (1.023–1.059)	<0.0001*	1.034 (1.018–1.051)	<0.0001*
**Large artery atherosclerosis (LAA)**				
Time (year)	0.999 (0.922–1.084)	0.99	0.972 (0.897–1.053)	0.48
Age	1.08 (1.05–1.111)	<0.0001*	1.086 (1.059–1.114)	<0.0001*
Black (vs white)	0.684 (0.334–1.403)	0.3	0.607 (0.312–1.178)	0.14
Other (vs white)	0.676 (0.266–1.717)	0.41	0.993 (0.316–3.121)	0.99
Current drinker	0.827 (0.473–1.448)	0.51	0.887 (0.505–1.559)	0.68
Smoker	1.888 (0.968–3.681)	0.06	1.553 (0.819–2.944)	0.18
Hypertension	1.442 (0.808–2.573)	0.22	1.04 (0.554–1.95)	0.9
Diabetes mellitus	2.009 (1.074–3.757)	0.029*	1.738 (0.849–3.558)	0.13
Hypercholesterolaemia	0.775 (0.399–1.506)	0.45	0.457 (0.231–0.906)	0.025*
Atrial fibrillation	1.169 (0.269–5.083)	0.83	4.123 (1.343–12.657)	0.013*
Myocardial infarction	1.719 (0.806–3.666)	0.16	2.084 (0.807–5.386)	0.13
TIA	0.93 (0.459–1.885)	0.84	1.531 (0.773–3.031)	0.22
Stroke unit admission (vs none)	1.429 (0.457–4.466)	0.54	1.068 (0.297–3.845)	0.92
Other medical wards (vs none)	1.327 (0.338–5.206)	0.68	1.281 (0.334–4.907)	0.72
Anticoagulant	0.724 (0.294–1.784)	0.48	1.157 (0.421–3.176)	0.78
Antiplatelet	0.423 (0.186–0.961)	0.042*	1.097 (0.39–3.09)	0.86
Thrombolysis	0.919 (0.392–2.155)	0.85	0.759 (0.312–1.848)	0.54
GCS	0.77 (0.625–0.949)	0.015*	0.771 (0.653–0.91)	0.002*
Failed swallowing	0.989 (0.467–2.095)	0.98	0.929 (0.499–1.732)	0.82
Urinary incontinence	0.998 (0.493–2.019)	0.99	1.408 (0.7–2.832)	0.34
BI (7d)	0.903 (0.839–0.971)	0.007*	0.929 (0.869–0.994)	0.032*
NIHSS	1.074 (1–1.153)	0.05	1.045 (0.978–1.117)	0.2
**Cardio–embolism (CE)**				
Time (year)	0.962 (0.922–1.003)	0.07	0.969 (0.939–1)	0.048*
Age	1.064 (1.049–1.079)	<0.0001*	1.057 (1.043–1.071)	<0.0001*
Black (vs white)	1.049 (0.7–1.571)	0.82	1.26 (0.851–1.868)	0.25
Other (vs white)	0.65 (0.35–1.205)	0.17	1.192 (0.765–1.855)	0.44
Current drinker	0.764 (0.536–1.09)	0.14	0.785 (0.603–1.022)	0.07
Smoker	1.772 (1.235–2.543)	0.002*	1.946 (1.225–3.091)	0.006*
Hypertension	1.065 (0.761–1.489)	0.71	0.981 (0.749–1.285)	0.89
Diabetes mellitus	1.184 (0.821–1.706)	0.37	1.134 (0.815–1.579)	0.46
Hypercholesterolaemia	0.962 (0.668–1.386)	0.84	0.947 (0.708–1.267)	0.71
Atrial fibrillation	1.106 (0.804–1.521)	0.54	1.283 (1.006–1.637)	0.045*
Myocardial infarction	0.886 (0.618–1.269)	0.51	1.308 (0.929–1.842)	0.12
TIA	1.031 (0.61–1.744)	0.91	1.096 (0.784–1.533)	0.59
Stroke unit admission (vs none)	0.84 (0.419–1.682)	0.62	0.741 (0.387–1.42)	0.37
Other medical wards (vs none)	1.603 (0.791–3.247)	0.19	1.03 (0.529–2.007)	0.93

Anticoagulant	0.842 (0.563–1.259)	0.4	0.982 (0.706–1.366)	0.91
Antiplatelet	0.62 (0.349–1.102)	0.11	0.767 (0.479–1.228)	0.27
Thrombolysis	0.765 (0.424–1.381)	0.38	0.854 (0.555–1.314)	0.47
GCS	0.923 (0.858–0.992)	0.03*	0.94 (0.895–0.987)	0.013*
Failed swallowing	1.141 (0.749–1.738)	0.54	1.13 (0.808–1.581)	0.48
Urinary incontinence	1.428 (0.935–2.181)	0.1	1.067 (0.75–1.519)	0.72
BI (7d)	0.965 (0.931–1.001)	0.06	0.933 (0.907–0.96)	<0.0001*
NIHSS	1.049 (1.019–1.08)	0.001*	1.03 (1.003–1.056)	0.027*
**Small vessel occlusion (SVO)**				
Time (year)	0.97 (0.917–1.027)	0.3	0.942 (0.888–0.999)	0.047*
Age	1.07 (1.052–1.089)	<0.0001*	1.057 (1.036–1.08)	<0.0001*
Black (vs white)	0.76 (0.513–1.126)	0.17	0.562 (0.342–0.923)	0.023*
Other (vs white)	1.141 (0.65–2.004)	0.65	0.448 (0.194–1.036)	0.06
Current drinker	1.035 (0.703–1.524)	0.86	0.594 (0.398–0.887)	0.011*
Smoker	1.493 (1.004–2.219)	0.048*	1.343 (0.852–2.119)	0.2
Hypertension	0.902 (0.62–1.313)	0.59	0.983 (0.651–1.484)	0.94
Diabetes mellitus	1.523 (1.028–2.257)	0.036*	0.945 (0.594–1.502)	0.81
Hypercholesterolaemia	1.033 (0.688–1.551)	0.88	1.308 (0.85–2.013)	0.22
Atrial fibrillation	1.582 (0.671–3.725)	0.29	2.221 (1.192–4.138)	0.012*
Myocardial infarction	1.332 (0.717–2.476)	0.36	2.775 (1.389–5.546)	0.004*
TIA	1.189 (0.725–1.95)	0.49	0.784 (0.389–1.577)	0.49
Stroke unit admission (vs none)	1.574 (0.911–2.72)	0.1	1.369 (0.801–2.339)	0.25
Other medical wards (vs none)	1.858 (0.993–3.475)	0.05	1.063 (0.577–1.961)	0.84
Anticoagulant	0.951 (0.459–1.973)	0.89	1.067 (0.56–2.035)	0.84
Antiplatelet	0.454 (0.245–0.84)	0.014*	0.669 (0.367–1.217)	0.19
Thrombolysis	1.381 (0.317–6.016)	0.67	0.687 (0.19–2.486)	0.57
GCS	1.03 (0.931–1.14)	0.57	0.869 (0.752–1.004)	0.06
Failed swallowing	1.557 (0.898–2.701)	0.12	1.678 (1–2.818)	0.05
Urinary incontinence	1.145 (0.702–1.868)	0.59	1.095 (0.683–1.757)	0.71
BI (7d)	0.935 (0.9–0.971)	0.0006*	0.927 (0.891–0.964)	0.0002*
NIHSS	1.014 (0.959–1.072)	0.63	1.007 (0.944–1.074)	0.83
**Undetermined aetiologies (UND)**				
Time (year)	0.97 (0.931–1.01)	0.14	0.99 (0.955–1.026)	0.58
Age	1.071 (1.057–1.085)	<0.0001*	1.056 (1.042–1.069)	<0.0001*
Black (vs white)	0.79 (0.566–1.103)	0.17	0.839 (0.599–1.175)	0.31
Other (vs white)	0.723 (0.45–1.162)	0.18	0.909 (0.541–1.526)	0.72
Current drinker	0.824 (0.622–1.091)	0.18	0.955 (0.728–1.254)	0.74
Smoker	1.513 (1.129–2.029)	0.006*	1.574 (1.172–2.114)	0.003*
Hypertension	0.906 (0.678–1.21)	0.5	0.98 (0.761–1.264)	0.88
Diabetes mellitus	1.421 (1.047–1.927)	0.024*	1.027 (0.769–1.37)	0.86
Hypercholesterolaemia	1.202 (0.87–1.661)	0.26	0.891 (0.666–1.193)	0.44
Atrial fibrillation	1.261 (0.873–1.821)	0.22	1.144 (0.813–1.609)	0.44
Myocardial infarction	0.765 (0.501–1.169)	0.22	1.563 (1.046–2.336)	0.029*
TIA	0.92 (0.605–1.396)	0.69	1.519 (1.083–2.13)	0.015*
Stroke unit admission (vs none)	1.233 (0.72–2.113)	0.45	0.812 (0.477–1.383)	0.44
Other medical wards (vs none)	1.993 (1.13–3.516)	0.017*	1.171 (0.707–1.939)	0.54
Anticoagulant	0.896 (0.536–1.499)	0.68	0.895 (0.592–1.352)	0.6
Antiplatelet	0.675 (0.458–0.995)	0.049*	0.558 (0.359–0.868)	0.012*
Thrombolysis	0.862 (0.505–1.471)	0.59	0.748 (0.466–1.199)	0.23
GCS	0.916 (0.862–0.973)	0.005*	0.864 (0.811–0.92)	<0.0001*
Failed swallowing	1.613 (1.167–2.231)	0.004*	1.379 (0.991–1.919)	0.06
Urinary incontinence	1.379 (0.97–1.961)	0.07	1.132 (0.821–1.563)	0.45
BI (7d)	0.959 (0.931–0.987)	0.005*	0.929 (0.904–0.954)	<0.0001*
NIHSS	1.031 (1.003–1.059)	0.031*	1.039 (1.012–1.067)	0.005*

Table B: Survival models for ISs by sex groups.

***** Denotes significant trends (p <0.05).

Abbreviations: BI, Barthel index; CI, confidence interval; GCS, Glasgow coma scale; NIHSS, National Institutes of Health Stroke Scale; and TIA, transient ischaemic attack.

	**Aged <65 years**		**Aged 65+**	
	HR (95% CI)	P Value	HR (95% CI)	P Value
**All ischaemic strokes (ISs)**				
Time (year)	0.985 (0.944–1.028)	0.49	0.975 (0.959–0.992)	0.003*
Age	1.052 (1.031–1.073)	<0.0001*	1.061 (1.053–1.069)	<0.0001*
Female (vs male)	0.811 (0.6–1.097)	0.17	0.865 (0.77–0.972)	0.015*
Black (vs white)	0.872 (0.618–1.229)	0.43	0.811 (0.694–0.946)	0.008*
Other (vs white)	0.889 (0.558–1.416)	0.62	0.806 (0.647–1.006)	0.06
Current drinker	0.737 (0.541–1.003)	0.05	0.857 (0.761–0.965)	0.011*
Smoker	1.816 (1.32–2.499)	0.0003*	1.522 (1.318–1.757)	<0.0001*
Hypertension	1.134 (0.826–1.557)	0.44	0.936 (0.826–1.059)	0.29
Diabetes mellitus	1.791 (1.284–2.499)	0.0006*	1.201 (1.049–1.376)	0.008*
Hypercholesterolaemia	0.899 (0.643–1.258)	0.54	0.94 (0.821–1.077)	0.37
Atrial fibrillation	0.997 (0.616–1.613)	0.99	1.425 (1.242–1.635)	<0.0001*
Myocardial infarction	1.168 (0.726–1.878)	0.52	1.248 (1.055–1.475)	0.01*
TIA	0.837 (0.509–1.375)	0.48	1.14 (0.973–1.337)	0.11
Stroke unit admission (vs none)	1.112 (0.645–1.917)	0.7	1.056 (0.844–1.321)	0.63
Other medical wards (vs none)	1.747 (0.983–3.103)	0.06	1.377 (1.087–1.743)	0.008*
Anticoagulant	0.91 (0.579–1.431)	0.69	0.975 (0.802–1.184)	0.8
Antiplatelet	0.568 (0.371–0.87)	0.011*	0.669 (0.542–0.826)	0.0004*
Thrombolysis	0.819 (0.474–1.414)	0.47	0.808 (0.641–1.018)	0.07
GCS	0.915 (0.856–0.978)	0.009*	0.921 (0.898–0.944)	<0.0001*
Failed swallowing	1.411 (0.946–2.104)	0.09	1.31 (1.129–1.52)	0.0004*
Urinary incontinence	1.078 (0.708–1.643)	0.73	1.223 (1.053–1.419)	0.008*
BI (7d)	0.938 (0.91–0.966)	<0.0001*	0.942 (0.93–0.955)	<0.0001*
NIHSS	1.053 (1.019–1.088)	0.002*	1.036 (1.023–1.049)	<0.0001*
**Large artery atherosclerosis (LAA)**				
Time (year)	1.005 (0.842–1.199)	0.96	0.982 (0.924–1.042)	0.54
Age	1.128 (1.015–1.253)	0.025*	1.066 (1.037–1.096)	<0.0001*
Female (vs male)	0.391 (0.094–1.623)	0.2	1.143 (0.77–1.697)	0.51
Black (vs white)	0.715 (0.166–3.084)	0.65	0.684 (0.41–1.14)	0.14
Other (vs white)	0.532 (0.064–4.393)	0.56	0.802 (0.379–1.696)	0.56
Current drinker	0.384 (0.108–1.37)	0.14	0.943 (0.627–1.418)	0.78
Smoker	5.744 (0.997–33.085)	0.05	1.34 (0.837–2.144)	0.22
Hypertension	2.29 (0.502–10.449)	0.28	1.161 (0.77–1.75)	0.48
Diabetes mellitus	3.415 (0.654–17.85)	0.15	1.843 (1.151–2.953)	0.011*
Hypercholesterolaemia	0.905 (0.171–4.786)	0.91	0.546 (0.33–0.903)	0.018*
Atrial fibrillation	7.032 (0.4–123.771)	0.18	1.723 (0.68–4.362)	0.25
Myocardial infarction	0.596 (0.069–5.152)	0.64	1.911 (1.042–3.505)	0.036*
TIA	0.6 (0.134–2.691)	0.51	1.451 (0.873–2.412)	0.15
Stroke unit admission (vs none)	3.682 (0.224–60.584)	0.36	1.212 (0.519–2.831)	0.66
Other medical wards (vs none)	19.981 (0.759–525.746)	0.07	1.203 (0.473–3.064)	0.7
Anticoagulant	1.11 (0.116–10.631)	0.93	0.905 (0.475–1.725)	0.76
Antiplatelet	0.402 (0.052–3.082)	0.38	0.66 (0.347–1.255)	0.21
Thrombolysis	0.531 (0.05–5.588)	0.6	0.855 (0.442–1.654)	0.64
GCS	0.597 (0.384–0.929)	0.023*	0.8 (0.702–0.911)	0.001*
Failed swallowing	1.882 (0.348–10.178)	0.46	1.012 (0.611–1.677)	0.96
Urinary incontinence	0.16 (0.022–1.174)	0.07	1.419 (0.867–2.324)	0.16
BI (7d)	0.872 (0.753–1.01)	0.07	0.939 (0.888–0.992)	0.027*
NIHSS	1.061 (0.874–1.29)	0.55	1.055 (1.002–1.11)	0.041*
**Cardio–embolism (CE)**				
Time (year)	0.958 (0.882–1.041)	0.31	0.969 (0.944–0.995)	0.022*
Age	1.075 (1.027–1.125)	0.002*	1.046 (1.031–1.061)	<0.0001*
Female (vs male)	0.872 (0.389–1.958)	0.74	0.802 (0.651–0.988)	0.038*
Black (vs white)	3.432 (1.38–8.538)	0.008*	0.974 (0.719–1.319)	0.86
Other (vs white)	1.276 (0.44–3.698)	0.65	0.795 (0.538–1.174)	0.25
Current drinker	0.651 (0.304–1.393)	0.27	0.82 (0.662–1.014)	0.07
Smoker	1.966 (0.927–4.169)	0.08	1.853 (1.359–2.527)	0.0001*
Hypertension	0.758 (0.345–1.662)	0.49	1.01 (0.81–1.259)	0.93
Diabetes mellitus	1.278 (0.526–3.105)	0.59	1.114 (0.86–1.441)	0.41
Hypercholesterolaemia	1.458 (0.628–3.385)	0.38	0.896 (0.703–1.14)	0.37
Atrial fibrillation	0.977 (0.433–2.202)	0.96	1.259 (1.029–1.539)	0.025*
Myocardial infarction	0.977 (0.415–2.302)	0.96	1.126 (0.868–1.461)	0.37
TIA	1.195 (0.385–3.714)	0.76	1.084 (0.817–1.438)	0.58
Stroke unit admission (vs none)	1.354 (0.12–15.286)	0.81	0.74 (0.459–1.191)	0.21
Other medical wards (vs none)	4.033 (0.377–43.142)	0.25	1.097 (0.668–1.802)	0.71
Anticoagulant	0.589 (0.237–1.466)	0.26	0.984 (0.745–1.298)	0.91

Antiplatelet	0.528 (0.184–1.518)	0.24	0.701 (0.475–1.034)	0.08
Thrombolysis	0.879 (0.284–2.721)	0.82	0.792 (0.557–1.125)	0.19
GCS	1.092 (0.946–1.26)	0.23	0.916 (0.878–0.957)	<0.0001*
Failed swallowing	1.83 (0.684–4.898)	0.23	1.087 (0.825–1.433)	0.55
Urinary incontinence	0.776 (0.299–2.012)	0.6	1.342 (1.004–1.794)	0.047*
BI (7d)	0.927 (0.852–1.008)	0.08	0.941 (0.919–0.964)	<0.0001*
NIHSS	1.083 (1.01–1.162)	0.026*	1.031 (1.01–1.053)	0.004*
**Small vessel occlusion (SVO)**				
Time (year)	0.896 (0.794–1.011)	0.07	0.976 (0.936–1.018)	0.26
Age	1.012 (0.958–1.07)	0.66	1.063 (1.043–1.083)	<0.0001*
Female (vs male)	0.742 (0.351–1.571)	0.44	0.881 (0.678–1.144)	0.34
Black (vs white)	1.058 (0.458–2.443)	0.9	0.667 (0.48–0.927)	0.016*
Other (vs white)	2.156 (0.8–5.813)	0.13	0.817 (0.492–1.357)	0.44
Current drinker	0.726 (0.316–1.666)	0.45	0.844 (0.64–1.113)	0.23
Smoker	4.09 (1.77–9.448)	0.001*	1.292 (0.941–1.773)	0.11
Hypertension	1.01 (0.439–2.323)	0.98	0.825 (0.619–1.101)	0.19
Diabetes mellitus	1.16 (0.466–2.89)	0.75	1.252 (0.922–1.699)	0.15
Hypercholesterolaemia	2.605 (1.087–6.245)	0.032*	1.043 (0.755–1.44)	0.8
Atrial fibrillation	0 (0–Inf)	1	2.128 (1.266–3.578)	0.005*
Myocardial infarction	2.66 (0.484–14.621)	0.26	1.655 (1.035–2.647)	0.035*
TIA	0.307 (0.063–1.487)	0.14	1.248 (0.829–1.878)	0.29
Stroke unit admission (vs none)	2.731 (0.851–8.767)	0.09	1.302 (0.863–1.965)	0.21
Other medical wards (vs none)	0.627 (0.128–3.065)	0.56	1.406 (0.882–2.243)	0.15
Anticoagulant	1.107 (0.143–8.595)	0.92	1.07 (0.609–1.879)	0.81
Antiplatelet	0.492 (0.142–1.709)	0.27	0.581 (0.366–0.923)	0.024*
Thrombolysis	0.83 (0.081–8.48)	0.88	0.992 (0.335–2.937)	0.99
GCS	0.943 (0.592–1.502)	0.8	0.981 (0.911–1.057)	0.61
Failed swallowing	2.002 (0.451–8.877)	0.36	1.601 (1.101–2.327)	0.014*
Urinary incontinence	2.365 (0.65–8.612)	0.19	1.061 (0.764–1.473)	0.72
BI (7d)	0.862 (0.79–0.942)	0.001*	0.942 (0.915–0.971)	0.0001*
NIHSS	0.931 (0.801–1.083)	0.36	1.03 (0.986–1.076)	0.19
**Undetermined aetiologies (UND)**				
Time (year)	0.999 (0.92–1.084)	0.98	0.981 (0.954–1.009)	0.19
Age	1.031 (0.996–1.067)	0.08	1.068 (1.054–1.083)	<0.0001*
Female (vs male)	1.498 (0.909–2.466)	0.11	0.785 (0.638–0.966)	0.023*
Black (vs white)	0.494 (0.263–0.93)	0.029*	0.835 (0.645–1.081)	0.17
Other (vs white)	0.608 (0.28–1.323)	0.21	0.847 (0.573–1.251)	0.4
Current drinker	0.815 (0.478–1.39)	0.45	0.904 (0.727–1.124)	0.37
Smoker	1.821 (1.067–3.109)	0.028*	1.62 (1.287–2.041)	<0.0001*
Hypertension	1.923 (1.125–3.286)	0.017*	0.841 (0.687–1.029)	0.09
Diabetes mellitus	1.998 (1.127–3.544)	0.018*	1.109 (0.881–1.396)	0.38
Hypercholesterolaemia	0.8 (0.438–1.463)	0.47	1.06 (0.835–1.346)	0.63
Atrial fibrillation	1.085 (0.496–2.378)	0.84	1.259 (0.974–1.627)	0.08
Myocardial infarction	1.656 (0.578–4.747)	0.35	1.033 (0.767–1.393)	0.83
TIA	0.928 (0.37–2.329)	0.87	1.254 (0.952–1.652)	0.11
Stroke unit admission (vs none)	1.126 (0.428–2.968)	0.81	1.041 (0.693–1.563)	0.85
Other medical wards (vs none)	1.424 (0.551–3.682)	0.47	1.511 (0.999–2.285)	0.05
Anticoagulant	0.644 (0.275–1.511)	0.31	0.909 (0.647–1.277)	0.58
Antiplatelet	0.409 (0.195–0.859)	0.02*	0.654 (0.467–0.916)	0.016*
Thrombolysis	0.755 (0.326–1.748)	0.51	0.777 (0.519–1.163)	0.22
GCS	0.873 (0.798–0.956)	0.003*	0.893 (0.852–0.937)	<0.0001*
Failed swallowing	1.094 (0.567–2.109)	0.79	1.433 (1.119–1.836)	0.005*
Urinary incontinence	1.696 (0.854–3.367)	0.13	1.215 (0.948–1.556)	0.12
BI (7d)	0.939 (0.892–0.988)	0.015*	0.943 (0.924–0.962)	<0.0001*
NIHSS	1.063 (1.014–1.114)	0.012*	1.035 (1.013–1.058)	0.002*

Table C: Survival models for ISs by age groups.

***** Denotes significant trends ($p < 0.05$).

Abbreviations: BI, Barthel index; CI, confidence interval; GCS, Glasgow coma scale; NIHSS, National Institutes of Health Stroke Scale; and TIA, transient ischaemic attack.

	Year Group				P Value for Trend
	2000–2003 (N=807)	2004–2007 (N=1117)	2008–2011 (N=637)	2012–2015 (N=567)	
**Medication use before stroke**					
Antihypertensive	371 (47.6)	590 (53.4)	207 (33.9)	205 (37.0)	<0.001*
Anti–diabetic	154 (19.2)	252 (22.6)	122 (19.4)	117 (21.0)	0.235
Cholesterol–lowering	91 (13.8)	298 (26.9)	235 (38.5)	212 (38.2)	<0.001*
Antiplatelet	256 (42.4)	426 (45.1)	228 (37.5)	169 (30.6)	<0.001*
Anticoagulant	30 (5.0)	43 (3.9)	27 (4.4)	38 (6.9)	0.06
**Medications at discharge**					
Antihypertensive	1.46 (0.50)	1.31 (0.46)	1.35 (0.48)	1.44 (0.50)	<0.001*
Anti–diabetic	1.22 (0.42)	1.17 (0.38)	1.19 (0.39)	1.19 (0.39)	0.791
Antiplatelet	1.12 (0.33)	1.09 (0.28)	1.75 (0.43)	1.78 (0.41)	<0.001*

Table D: Trends in medication use among patients with IS.

Data are counts (percentages). P–values were obtained from the Cochran–Armitage tests for trends.

***** Denotes significant trends (*p* <0.05).

	Ischaemic Stroke Subtype					P Value
	LAA (N=351)	CE (N=815)	SVO (N=791)	UND (N=1089)	OTH (N=82)	
**Medication use before stroke**						
Antihypertensive	159 (46.0)	376 (47.7)	349 (45.1)	466 (44.0)	23 (28.4)	<0.0001*
Anti–diabetic	75 (21.5)	145 (18.0)	171 (21.7)	245 (22.7)	9 (11.1)	0.81
Cholesterol–lowering	107 (32.5)	231 (30.4)	200 (27.0)	288 (28.0)	10 (13.2)	<0.0001*
Antiplatelet	124 (39.9)	337 (47.2)	253 (37.9)	349 (36.4)	16 (27.6)	0.1
Anticoagulant	5 (1.6)	74 (9.9)	15 (2.1)	38 (3.8)	6 (8.2)	<0.0001*
**Medications at discharge**						
Antihypertensive	1.40 (0.49)	1.29 (0.46)	1.42 (0.49)	1.34 (0.47)	1.26 (0.44)	
Anti–diabetic	1.19 (0.40)	1.13 (0.34)	1.21 (0.41)	1.20 (0.40)	1.08 (0.28)	
Antiplatelet	1.59 (0.49)	1.54 (0.50)	1.63 (0.48)	1.57 (0.50)	1.43 (0.50)	<0.0001*

Table E: Medication use among patients with ISs by aetiological subtypes.

Data are counts (percentages).

***** Denotes significant trends (*p* <0.05).

Characteristic	BI at 3 months		P Value
	Completed (N=1766)	Missing (N=827)	
Age (mean (SD))	69.65 (14.00)	67.89 (15.17)	0.002*
Female	846 (47.9)	381 (46.1)	0.37
Ethnic group			0.16
White	1143 (64.7)	518 (62.6)	
Black	491 (27.8)	230 (27.8)	
Other/Unknown	134 (7.6)	80 (9.7)	
Current drinker	934 (52.9)	436 (52.7)	0.95
Smoker	498 (28.2)	249 (30.1)	0.31
Hypertension	1173 (66.4)	552 (66.7)	0.93
Diabetes mellitus	427 (24.2)	182 (22)	0.21
Hypercholesterolaemia	547 (31)	236 (28.5)	0.18
Atrial fibrillation	263 (14.9)	126 (15.2)	0.86
Myocardial infarction	152 (8.6)	79 (9.6)	0.49
TIA	192 (10.9)	86 (10.4)	0.73
Antihypertensive	804 (45.5)	359 (43.4)	0.32
Anti–diabetic	394 (22.3)	168 (20.3)	0.25
Antiplatelet	685 (38.8)	330 (39.9)	0.62
Anticoagulant	72 (4.1)	37 (4.5)	0.76
Cholesterol–lowering	535 (30.3)	231 (27.9)	0.22
Urinary incontinence	493 (27.9)	212 (25.6)	0.21
Swallow test (fail)	367 (20.8)	177 (21.4)	0.78
GCS <13	212 (12)	106 (12.8)	0.62
BI <15 (7d)	775 (43.9)	312 (37.7)	0.003*

Table F: Differences between IS patients with complete BI at 3 months and those without.

Data are count (%) unless otherwise indicated.

***** Denotes significant trends (*p* <0.05).

Abbreviations: BI, Barthel index; CI, confidence interval; GCS, Glasgow coma scale; SD, standard deviation; and TIA, transient ischaemic attack.

	Adjusted Disability Rates (mRS≥3) †				Adjusted Rate Ratio per Year (95% CI) ‡	P Value for Trend §
	2000–2003	2004–2007	2008–2011	2012–2015		
**At initial assessment (7d)**						
Overall ISs	58.1	58.4	48.5	45.3	0.979 (0.968–0.99)	<0.0001*
By ethnicity						
White	59.9	59.5	50.4	45.2	0.977 (0.964–0.991)	<0.0001*
Black	53.0	55.3	39.9	43.9	0.98 (0.958–0.999)	0.006*
By sex						
Male	51.2	50.3	37.9	32.3	0.965 (0.948–0.982)	<0.0001*
Female	64.1	65.0	58.0	58.8	0.99 (0.975–1.005)	0.048*
By age group						
<55 y	35.9	35.9	28.0	29.3	0.977 (0.952–0.999)	0.041*
55+ y	66.1	66.2	55.5	50.6	0.979 (0.966–0.992)	<0.0001*
By TOAST subtypes						
LAA	74.1	63.4	58.2	55.0	0.981 (0.947–0.999)	0.021*
CE	69.9	68.6	61.2	56.1	0.982 (0.963–0.999)	0.002*
SVO	38.5	41.2	30.0	24.5	0.965 (0.938–0.992)	0.002*
UND	63.2	61.4	50.6	48.4	0.978 (0.959–0.997)	<0.0001*
**At 3 months post–stroke**						
Overall ISs	43.6	38.9	38.1	35.1	0.984 (0.969–0.999)	0.002*
By ethnicity						
White	43.7	37.6	37.2	37.0	0.986 (0.967–0.999)	0.033*
Black	43.2	38.1	34.7	30.4	0.978 (0.948–0.999)	0.01*
By sex						
Male	39.3	31.4	29.6	29.0	0.977 (0.954–0.999)	0.004*
Female	47.8	46.6	46.3	41.0	0.99 (0.968–1.011)	0.11
By age group						
<55 y	32.8	27.3	27.2	21.7	0.972 (0.939–0.999)	0.015*
55+ y	48.1	43.3	41.6	40.2	0.986 (0.968–0.999)	0.011*
By TOAST subtypes						
LAA ¶	71.8	38.3	47.5	35.4	0.962 (0.92–1.007)	0.002*
CE	52.0	47.7	45.2	45.3	0.992 (0.963–1.022)	0.16
SVO ¶	29.1	27.4	24.5	22.6	0.978 (0.945–1.013)	0.13
UND	46.8	43.7	42.6	37.4	0.982 (0.955–1.011)	0.047*

Table G: Trends in functional dependence (mRS ≥3) at initial assessment and 3 months after the first-ever IS.

Rankin scale was derived from Barthel index score (Wolfe, Taub et al. 1991).

***** Denotes significant trends (*p* <0.05).

† Unless otherwise indicated, figures are adjusted for demographic variables as appropriate. Adjusted rates for each time cohort were obtained by multiplying the observed rate for the reference period (2000–2003) by the corresponding rate ratios for the later periods from a model evaluating time cohorts as a categorical variable.

‡ Adjusted risk ratios were determined with a model evaluating calendar year as a continuous variable.

§ P values were obtained from the Cochran-Armitage tests for trend.

¶ Unadjusted because of small number of events.

Abbreviations: CE, cardio-embolism; CI, confidence interval; IS, ischaemic stroke; LAA, large-artery atherosclerosis; mRS, modified Rankin Scale; SVO, small-vessel occlusion; TOAST, Trial of ORG 10172 in Acute Stroke Treatment; UND, undetermined aetiologies.

References:

Wolfe, C. D., N. A. Taub, E. J. Woodrow and P. G. Burney (1991). "Assessment of scales of disability and handicap for stroke patients." Stroke 22(10): 1242-1244.
