# Supplementary material for: Long-term trends in death and dependence after ischaemic strokes: A retrospective cohort study using the South London Stroke Register (SLSR)
Source: PLoS Med. 2020 Mar 12;17(3):e1003048. doi: 10.1371/journal.pmed.1003048 (PMC7067375; doi:10.1371/journal.pmed.1003048)
Supplement: S2 Appendix — (PDF) [file pmed.1003048.s003.pdf]

8441

SOUTH LONDON STROKE REGISTER

ANNUAL FOLLOW UP

ID Number ☐☐☐☐

Thank you for taking time to complete this questionnaire. It will help us to know how you are getting on since your stroke.

Please read the following guidelines before beginning:

- **Use blue or black ink for filling in the questionnaire.**
- **Answer all questions. We are well aware that some questions might not seem relevant to you personally, but please try to answer them all as best you can.**
- **You should complete the form yourself. However, if you are unable to then a carer or relative may help you.**
- **Most questions require you to select your answer from choices given to you. To do this please place a cross in the box beside the *<u>one</u>* choice which best describes your situation/feelings, as shown in the example below :**

Q. Is the sky blue?

☒ Yes ☐ No

1. Is anyone helping you complete this questionnaire?

☐ I am answering on my own

☐ I am a carer/family member/friend answering on his/her behalf.

2. What is today's date?

DAY ☐☐ / **MONTH** ☐☐ / **YEAR** ☐☐☐☐

3. What is your date of birth?

DAY ☐☐ / **MONTH** ☐☐ / **YEAR** ☐☐☐☐

4. Where do you live?

☐ Private household alone (including private/council rented accommodation)

☐ Private household with others (including private/council rented accommodation)

☐ Sheltered home

☐ Care home

☐ Other *Specify:* ______________________

5. What is your current employment status?

☐ Full time employed *(more than 30hrs/wk)*

☐ Part time employed *(less than 30hrs/wk)*

☐ Retired

☐ Carer for home/family/dependents

☐ Unemployed

☐ Unable to work due to disability/ill-health

SOUTH LONDON STROKE REGISTER
ANNUAL FOLLOW UP

ID Number ☐☐☐☐

6a. Have you had another stroke in the last year?

☐ Yes ☐ No ☐ I don't know

6b.Have you been readmitted to hospital since the last follow up?

☐ Yes ☐ No → Go to question 7

6c. What was the name of the hospital?

☐

6d. Were you in hospital because you had had another stroke?

☐ Yes ☐ No ☐ I don't know

7. In the last year have you experienced any of the following symptoms?

**a. New visual problems**	☐ Yes	☐ No	☐ I don't know
**b. New speech problems**	☐ Yes	☐ No	☐ I don't know
**c. New weakness of arms/legs**	☐ Yes	☐ No	☐ I don't know

7d.If yes to any of the above, did you see your GP about the new symptoms?

☐ Yes ☐ No

8a. In the last 2 weeks, have you required help from another person for everyday activities (such as making a cup of tea)?

☐ Yes ☐ No → Go to question 9

8b. If yes, who did you receive most help from?

☐ Home help or carer ☐ Son ☐ Friend

☐ Spouse/partner ☐ Other relative ☐ Voluntary Organisation

☐ Daughter ☐ Other professional care (paid/unpaid)

☐ Other
Specify ______________________

9. Has a member of your family given up work since the stroke to care for you?

☐ Yes ☐ No

SOUTH LONDON STROKE REGISTER
ANNUAL FOLLOW UP

ID Number

10. Are you still in hospital, a nursing home or a residential home?

☐ Yes → Go to question 18 ☐ No

11. Do your friends and family help you (at least once a week) with any of the following?

a. Cleaning the house ☐ Yes ☐ No

b. Preparing meals ☐ Yes ☐ No

c. Shopping ☐ Yes ☐ No

d. Having a bath or shower ☐ Yes ☐ No

12. In the last week have you had any meals on wheels?

☐ Yes ☐ No

→ **How many times?**

13. In the last week have you had any home help?

☐ Yes ☐ No

→ **How many times?**

14. In the last week have you attended a day centre?

☐ Yes ☐ No

→ **How many times?**

15. In the last week have you attended a day hospital?

☐ Yes ☐ No

→ **How many times?**

16. In the last week have you had a district nurse visit you?

☐ Yes ☐ No

→ **How many times?**

17. In the last year, have you been admitted to a respite home for a short time to give yourself and your carer a rest?

☐ Yes ☐ No

SOUTH LONDON STROKE REGISTER
ANNUAL FOLLOW UP

ID Number ☐☐☐☐

18a. Have you had any physiotherapy in the last year?

☐ Yes ☐ No → Go to question 19

18b. Do you still have this therapy?

☐ Yes ☐ No

19a. Have you had any occupational therapy in the last year?

☐ Yes ☐ No → Go to question 20

19b. Do you still have this therapy?

☐ Yes ☐ No

20a. Have you had any speech or language therapy in the last year?

☐ Yes ☐ No → Go to question 21

20b. Do you still have this therapy?

☐ Yes ☐ No

21a. Have you see a psychologist in the last year?

☐ Yes ☐ No → Go to question 22

21b. Do you still see them?

☐ Yes ☐ No

22a. Have you seen a GP in the last year?

☐ Yes ☐ No → Go to question 23

22b. Have you seen them in the last month?

☐ Yes ☐ No

→ **How many times?** ☐☐

23. Do you have weakness of an arm or a leg due to your stroke?

☐ Yes ☐ No ☐ I don't know

24. Do you have any difficulties with your speech due to your stroke?

☐ Yes ☐ No ☐ I don't know

25. Do you have any trouble swallowing due to your stroke?

☐ Yes ☐ No ☐ I don't know

SOUTH LONDON STROKE REGISTER
ANNUAL FOLLOW UP

ID Number ☐☐☐☐

26. Have you ever been diagnosed with any of the following?

**Depression**	☐ Yes	☐ No	☐ I don't know
**High blood pressure**	☐ Yes	☐ No	☐ I don't know
**High Cholesterol**	☐ Yes	☐ No	☐ I don't know
**Diabetes**	☐ Yes	☐ No	☐ I don't know
**Atrial fibrilation** (Irregular heartbeat)	☐ Yes	☐ No	☐ I don't know
**Angina**	☐ Yes	☐ No	☐ I don't know
**Peripheral vascular disease** (narrowing of arteries in legs)	☐ Yes	☐ No	☐ I don't know
**Epilepsy**	☐ Yes	☐ No	☐ I don't know
**Heart attack** (Myocardial infarction)	☐ Yes	☐ No	☐ I don't know

Was it within the last month?
☐ Yes ☐ No

27a. Do you smoke?

☐ Yes i am a smoker

☐ I am an ex-smoker

☐ I have never smoked → Go to question 27e

27b. If you are a smoker, how much do you smoke a day?

Cigarettes (number) ☐☐☐

Tobacco (grams) ☐☐

Cigars (number) ☐☐

27c. Do you smoke e-cigarettes?

☐ Yes ☐ No

27d. If you are an ex-smoker, have you given up in the last year?

☐ Yes ☐ No

27e. Have you taken any recreational drugs in the last year?

☐ Yes ☐ No

27f. If yes, which one(s)?

☐ Opiates (e.g. Opium/Heroin) ☐ Ketamine ☐ Cannabis

☐ Cocaine ☐ Ecstasy ☐ Other *Specify* ____________________

SOUTH LONDON STROKE REGISTER

ANNUAL FOLLOW UP

ID Number ☐☐☐☐

28a. Do you drink any alcohol?

☐ Yes ☐ No → Go to question 29

28b. How much do you drink a week?

Beer (pints) ☐☐☐

Spirits (glasses) ☐☐☐ ☐ I don't drink every week

Wine (glasses) ☐☐☐

29. Do you feel that you have made a complete recovery from the stroke?

☐ Yes ☐ No

30. Have you had any written information about preventing further strokes?

☐ Yes ☐ No

31a. Are you currently on any medication?

☐ Yes ☐ No → Go to the next page

31b. Please list all the medications you are currently taking in the spaces provided below:

	Name of Medication
A.	
B.	
C.	
D.	
E.	
F.	
G.	
H.	
I.	
J.	
K.	
L.	
M.	
N.	
O.	

8441

SOUTH LONDON STROKE REGISTER

ANNUAL FOLLOW UP

ID Number | | | | |

Please answer yes or no to the following questions about support you receive from those around you

1. If you needed help, do you have anyone (e.g. friends, neighbours, family) that you can turn to?

☐ Yes ☐ No

2. Do you have somebody (e.g. friends, neighbours, family) who shows that they care about you? ☐ Yes ☐ No

3. Do you see as much of your neighbours as you would like?

☐ Yes ☐ No ☐ I don't have any

4. Do you see as much of your relatives as you would like?

☐ Yes ☐ No ☐ I don't have any

5. Do you see as much of your friends as you would like?

☐ Yes ☐ No ☐ I don't have any

On the following two pages are some questions about your ability to look after yourself. They may not all seem to apply to you but please answer them all by selecting *one* option which you feel best describes your situation .

1. In the bath or shower, do you:

☐ manage on your own?

☐ need help getting in and out?

☐ need other help?

☐ never have a bath or shower?

☐ need to be washed in bed?

2. Can you climb stairs at home:

☐ without anyone's help?

☐ with someone encouraging you?

☐ with someone carrying your frame?

☐ with physical help?

☐ not at all?

☐ don't have stairs?

3. Do you get dressed:

☐ without any help?

☐ just with help with buttons?

☐ with someone helping you most of the time?

4. Do you walk indoors:

- ☐ without anyones help or with a frame?
- ☐ with one person watching over you?
- ☐ with one person helping you
- ☐ with more than one person helping you?
- ☐ not at all?
- ☐ or do you use a wheelchair independently(e.g. round corners)?

5. Do you move from bed to chair:

- ☐ on your own?
- ☐ with a little help from one person?
- ☐ with a lot of help from one or more people?
- ☐ not at all?

6. Do you eat food:

- ☐ without any help?
- ☐ with some help(such as cutting food or spreading butter)?
- ☐ with more help?

7. Do you use the toilet or commode:

- ☐ without anyone's help?
- ☐ with some help but can do somethings?
- ☐ with quite a lot of help?

8. Do you brush your hair and teeth, wash your face and shave:

- ☐ without help?
- ☐ with help?

9. Do you lose control of your bladder? (are you incontinent of urine?):

- ☐ never
- ☐ less than once a week
- ☐ less than once a day
- ☐ more often
- ☐ or do you have a catheter managed for you?

10. Do you lose control of your bowel movements? (Do you soil yourself?):

- ☐ never
- ☐ occasional accident
- ☐ all the time

SOUTH LONDON STROKE REGISTER
ANNUAL FOLLOW UP

ID Number ☐☐☐☐

We are interested in finding out how often you carry out some activities. As you will see the first pageis about activities during the last *3 months* and over the page ask about the last *6 months*.
Please remember to select one box only for each question.

In the last 3 months how often have you carried out these activities?

1. Preparing main meals (not just a snack)
☐ Never ☐ Less than once a week ☐ 1 or 2 times a week ☐ Most days

2. Washing up (Do all after one meal or share equally with another person)
☐ Never ☐ Less than once a week ☐ 1 or 2 times a week ☐ Most days

Over the last 3 months how often have you carried out these activities?

3. Washing clothes (e.g. loading and unloading washing machine)
☐ Never ☐ Only once or twice ☐ 1 to 4 times a month ☐ At least once a week

4. Light housework (e.g. dusting, or tidying small objects)
☐ Never ☐ Only once or twice ☐ 1 to 4 times a month ☐ At least once a week

5. Heavy housework (e.g. hoovering, or making beds)
☐ Never ☐ Only once or twice ☐ 1 to 4 times a month ☐ At least once a week

6. Local shopping
☐ Never ☐ Only once or twice ☐ 1 to 4 times a month ☐ At least once a week

7. Social occasions (including going to church)
☐ Never ☐ Only once or twice ☐ 1 to 4 times a month ☐ At least once a week

8. Walking outside for over 15 minutes
☐ Never ☐ Only once or twice ☐ 1 to 4 times a month ☐ At least once a week

9. Taking part in a hobby activity
☐ Never ☐ Only once or twice ☐ 1 to 4 times a month ☐ At least once a week

10. Going on a bus or driving a car
☐ Never ☐ Only once or twice ☐ 1 to 4 times a month ☐ At least once a week

SOUTH LONDON STROKE REGISTER
ANNUAL FOLLOW UP

ID Number ☐☐☐☐

In the last 6 months how often have you carried out the following activities?

11. Travel outings or car rides (travel for pleasure, not just for routine trips)

☐ Never ☐ Only once or twice ☐ 1 to 2 times a month ☐ At least once a week

12.Gardening

☐ Never ☐ Light (e.g. occasional weeding) ☐ Moderate (regular work) ☐ All necessary (includes heavy digging)

13. Household or car maintenance

☐ Never ☐ Light (e.g. small repairs) ☐ Moderate (e.g. painting) ☐ All necessary

14. Reading books (not just magazines)

☐ Never ☐ One in 6 months ☐ Less than 1 a fortnight ☐ More than 1 a fortnight

15. Paid work

☐ None ☐ Up to 10hrs a week ☐ 10-30hrs a week ☐ More than 30hrs a week

The following questions ask for your views about your health, how you feel and how well you are able to do your usual activities.
If you are unsure about how to answer any questions please give the best answer you can and make any of your own comments if you like. Do not spend too much time in answering as your immediate response is likely to be the most accurate.

1. In general, would you say your health is:

☐ Excellent ☐ Very Good ☐ Good ☐ Fair ☐ Poor

2. Health and daily activities. The following questions are about activities you might do during a particular day. Does your health limit you in these activities? If so, how much?

A. Moderate activities (such as moving a table,pushing a vacuum, bowling or playing golf)

☐ Yes, limited a lot ☐ Yes, limited a little ☐ No, not limited at all

B. Climbing several flights of stairs

☐ Yes, limited a lot ☐ Yes, limited a little ☐ No, not limited at all

3. During the past 4 weeks, have you had any of the following problems with your work or other regular daily activities as a result of your physical health? (Please answer Yes or No to each question)

A. Accomplished less than you would like

☐ Yes ☐ No

B. Were limited in the kind of work or other activities

☐ Yes ☐ No

ID Number [ ][ ][ ][ ]

4. During the past 4 weeks, have you had any of the following problems with your work or other regular daily activities as a result of any emotional problems (such as feeling depressed or anxious)? (Please answer Yes or No to each question)

A. Accomplished less than you would like

☐ Yes ☐ No

B. Didn't do work or activities as carefully as usual

☐ Yes ☐ No

5. During the past 4 weeks how much did pain interfere with your normal work (including work both outside the home and housework)? (Please tick one box)

☐ Not at all ☐ A little bit ☐ Moderately ☐ Quite a bit ☐ Extremely

6. These questions are about how you feel and how things have been with you during the past month. For each question, please indicate the one answer that comes closest to the way you have been feeling. (Please tick one box)

How much time during the last month:

A. Have you felt calm and peaceful?

☐ All of the time ☐ A good bit of the time ☐ A little of the time
☐ Most of the time ☐ Some of the time ☐ None of the time

B. Did you have a lot of energy?

☐ All of the time ☐ A good bit of the time ☐ A little of the time
☐ Most of the time ☐ Some of the time ☐ None of the time

C. Have you felt downhearted and low?

☐ All of the time ☐ A good bit of the time ☐ A little of the time
☐ Most of the time ☐ Some of the time ☐ None of the time

D. Has your health limited your social activities?

☐ All of the time ☐ A good bit of the time ☐ A little of the time
☐ Most of the time ☐ Some of the time ☐ None of the time

This questionnaire is designed to help us know how you feel. Please give the reply which comes closest to how you have been feeling in the past week. Don't take too long over your replies: your immediate reaction to each item will probably be more accurate than a long thought out response.

1. I feel tense or 'wound up':

☐ most of the time ☐ a lot of the time ☐ occasionally ☐ not at all

2. I feel as if I am slowed down:

☐ nearly all the time ☐ very often ☐ sometimes ☐ not at all

8441

SOUTH LONDON STROKE REGISTER
ANNUAL FOLLOW UP

ID Number | | | | |

3. I still enjoy the things I used to:

- ☐ definitely as much
- ☐ not quite as much
- ☐ only a little
- ☐ hardly at all

4. I get a sort of frightened feeling like butterflies in my stomach:

- ☐ not at all
- ☐ occasionally
- ☐ quite often
- ☐ very often

5. I get a sort of frightened feeling as if something awful is about to happen:

- ☐ very definitely and quite badly
- ☐ yes, but not too badly
- ☐ a little, but it doesn't worry me
- ☐ not at all

6. I have lost interest in my appearance:

- ☐ definitely
- ☐ I dont take as much care as I should
- ☐ I may not take as much care as I should
- ☐ I take just as much care as ever

7. I can laugh and see the funny side of things:

- ☐ as much as I always could
- ☐ definitely not so much now
- ☐ not quite so much now
- ☐ not at all

8. I feel restless as if I have to be on the move:

- ☐ very much indeed
- ☐ quite a lot
- ☐ not very much
- ☐ not at all

9. Worrying thoughts go through my mind:

- ☐ a great deal of the time
- ☐ a lot of the time
- ☐ from time to time
- ☐ only occasionally

10. I look forward with enjoyment to things:

- ☐ as much as I ever did
- ☐ definitely less than I used to
- ☐ rather less than I used to
- ☐ hardly at all

11. I feel cheerful:

- ☐ not at all
- ☐ not often
- ☐ sometimes
- ☐ most of the time

12. I get sudden feelings of panic:

- ☐ very often indeed
- ☐ quite often
- ☐ not very often
- ☐ not at all

13. I can sit at ease and feel relaxed:

- ☐ definitely
- ☐ usually
- ☐ not often
- ☐ not at all

14. I can enjoy a good book or radio or tv programme:

- ☐ often
- ☐ sometimes
- ☐ not often
- ☐ very seldom

Thank you for taking time to complete this questionnaire
